# Surgical cancellation and changes in postoperative level of care among high and moderate risk surgical patients in the UK

**DOI:** 10.1177/17511437261495043

**Published:** 2026-10-12

**Authors:** Joanna K. L. Wong, Rosanna Sasson, Christopher Oddy, Pan-London Perioperative Audit and Research Network Committee, The REPACC Study Investigators

**Affiliations:** 1 Royal Free NHS Trust, London, UK; 2 Barts Health NHS Trust, London, UK; 3 Bloomsbury Institute of Intensive Care Medicine, University College London, UK

**Keywords:** high-risk surgery, enhanced care, intermediate care, critical care, cancellation, escalation, de-escalation, care levels

## Abstract

**Background:**

The clinical and administrative pressures leading to cancellation of surgery are well established. However, it is not known whether the same factors lead to a change in patients’ planned postoperative destination and level of care.

**Methods:**

We conducted a secondary analysis of the REPACC dataset; a nationwide, retrospective, observational study examining the provision of enhanced and critical care services in the UK. The reasons for cancellation and changes to the level of care received postoperatively were ascertained from free-text data.

**Results:**

Our analysis included 5990 patients from 65 sites across the UK, of which 539 (9.6%) had their surgery cancelled. The most common reason for surgical cancellation was a lack of resources (51.0%), primarily bed shortages (37.1%). Among the 5451 patients who proceeded with surgery, 81.3% received the anticipated level of care postoperatively, 3.9% required escalation and 14.8% were de-escalated to a lower level of care. Changes to the anticipated level of care occurred most often among patients initially triaged to enhanced care. Clinical reasons were the major cause of escalation (53.3%) and de-escalation (61.2%) postoperatively with bed shortages accounting for a minority. Patients were commonly de-escalated following a postoperative assessment (21.8%).

**Conclusion:**

Cancellation of surgery in the UK is primarily a consequence of resource scarcity, whereas clinical reasons account for the majority of those re-allocated to different levels of care postoperatively. Future work should explore the clinical and health economic impacts of this dynamic bed management with particular focus on the role of enhanced care as an interface between critical care and the ward.

## Introduction

Cancellation of elective surgery represents a major operational inefficiency in healthcare systems worldwide. This subject has been studied extensively within the UK National Health Service (NHS) due to the availability of comprehensive national data, largely stemming from a demand by service users for transparency in a publicly owned system, thus acting as a case study for similarly developed perioperative structures globally.^1–3^ Analyses show that approximately half of the elective surgical cancellations in the UK occur due to avoidable non-clinical reasons, particularly as a result of bed shortages and staff unavailability.^1,2^ Resource pressures are known to drive cancellations, however, it is not known whether they also drive changes in planned postoperative destination between different levels of care.

Determining the appropriate postoperative destination for each individual following elective surgery, frequently a decision between ward-based care or critical care admission, is an important aspect of perioperative planning. Over- and under-triage of patients with a high-risk of morbidity following surgery influences the quality of care delivered by a service and impacts resource use and departmental strain.^4,5^ Despite the importance of this aspect of patient care and the forethought put into each triage decision, changes to the anticipated postoperative level of care occur in roughly a quarter of surgical patients—often in the context of capacity constraints that might otherwise lead to cancellation.^6,7^

The impact of routine postoperative critical care admission on outcomes after elective surgery remains debated.^4,8–10^ While some cohorts show a mortality benefit,^6^ critical care admission may expose patients to avoidable harm imparted by sleep deprivation, unnecessary intervention and loss of autonomy brought about by the presence of continuous monitoring and the physical environment.^8,9^ It is estimated that less than half of those admitted to critical care after elective surgery require the invasive therapies offered in such an environment and yet are exposed to the associated risks.^8^ Furthermore, in resource-constrained systems, over-triage increases critical care workload and may indirectly contribute to surgical cancellation.^1,4^ Thus, in the absence of a clear mortality benefit associated with routine critical care admission, the practice of de-escalating patients to lower levels of care postoperatively may reflect appropriate risk management by clinicians. On the other hand, patients referred for postoperative care within critical care that are instead admitted to the ward exhibit high rates of rapid response Medical Emergency Team (MET) activation.^11^ From this group, those that undergo clinical deterioration requiring admission to critical care have increased mortality compared to those admitted directly to higher levels of care.^12^ Additionally, postoperative care delivery varies considerably between institutions, where some have distinct units for each level of support and others deliver multiple levels of care within the same unit allowing for fluid movements between them. Further variability complicates comparisons as the scope of monitoring and interventions for each care level may vary between sites. These observations highlight the potential conflicts between patient safety and resource efficiency, underscoring the importance of identifying which factors drive decision making when re-allocating patients to different levels of care after surgery.

Presently, little is known about the circumstances that lead to changes in planned postoperative destination; to what extent these overlap with those relevant to surgical cancellation; and how the widespread adoption of enhanced care pathways has impacted decision making in the postoperative period.^13^ Understanding this could help reduce patient harm, improve capacity planning and inform standards of care in postoperative care planning. Therefore, we aim to provide an updated analysis of the incidence of cancellation and changes in anticipated postoperative care level among moderate- and high-risk surgical patients in the UK. Furthermore, we aim to explore the reasons behind these linked phenomena in a contemporary cohort and delineate, for the first time, the role of enhanced care within this system of dynamic risk management.

## Methods

### Study design and ethical approval

This is a secondary analysis of the REPACC (Retrospective Evaluation of Postoperative Alternatives to Critical Care) study. Kingston and Richmond NHS Foundation Trust provided study sponsorship and Caldicott Guardian approval. Ethical review was not required based on the NHS Health Research Authority (HRA) tool.^14^ This report followed the Strengthening the Reporting of Observational studies in Epidemiology (STROBE) guidelines.^15^

### Data collection

REPACC was a national, multicentre, retrospective, observational study, delivered through anaesthesia resident research networks in the UK over 3 months from September to November 2023. Full methodological details have been reported previously,^16^ and data collection guides and case report forms are provided as Supplemental Appendices B–D. This analysis was performed using patient level data from the wider REPACC dataset including all adults electively referred for postoperative care within enhanced care or critical care at participating sites. Patients planned for ward-based care, undergoing emergency surgery, elective cardiothoracic, neurosurgical or obstetric procedures were excluded, as were patients referred for enhanced or critical care intra- or postoperatively. All datasets were linked to site-level surveys completed by local investigators describing local perioperative pathways involving enhanced and critical care. Data submitted by local investigators underwent quality checks and cross-verification, through data validation rules and sense checking analyses built-in to the database, with queries returned to investigators until resolved. A full description of our quality assurance processes is available online.^16^

All data were stratified according to the UK Intensive Care Society critical care levels system where level 3 represents intensive care; level 2, high dependency care; level 1, enhanced care, known alternatively as intermediate care in Europe and the United States; and level 0, care within standard surgical wards.^17^ The proposed and actual postoperative level of care was collected according to this system of stratification. Where possible this was conducted using data derived from bed management software. For all patients this information was cross-checked manually by local investigators. The level of care was reported to reflect the facility each patient was admitted to, as opposed to the therapies received within that facility. For example, a patient admitted to intensive care (level 3) that received monitoring only (level 1) would be recorded as admitted to level 3. At sites where different levels of care were housed within the same physical spaces and the level of care of the bedspace they were admitted to could not be accurately determined, patients were assigned the highest level of care deliverable within the admitting facility. Cancellation of surgery was identified through surgical scheduling software and similarly cross-verified. The reasons for cancellation and changes in the anticipated postoperative level of care were identified through clinical documentation. Reasons were recorded as free text alongside a binary classification sub-categorising changes and cancellations as related to bed capacity or not.

### Data analysis and statistics

The reasons for a change in postoperative location and cancellation were submitted in free-text format by local investigators. An inductive approach was used to group responses into clinically meaningful categories based on recurring themes. Categories were refined iteratively to ensure consistency across the dataset. Where possible, free-text responses were mapped to existing predefined categories. For comparison, the reasons for a change in planned care were grouped into clinical reasons; administrative reasons; personal reasons; and reasons related to a lack of resources. The primary surgical speciality caring for each patient was also submitted in free-text format and was mapped to established surgical categories. Where information regarding surgical speciality was not available, if present and unambiguous, procedural data was used to delineate this parameter. Anonymised data linking free-text descriptions to cancellation and level of care change categorisations are available in the Supplemental Appendix E.

Categorisations of free-text data were conducted independently by two authors (JW/CO) and assessed for concordance. The final dataset was assessed for accuracy and approved by all authors. Continuous variables were reported as medians and interquartile ranges (IQR) and discrete data as counts and percentages. All analyses were conducted using Python version 3.10.6 (Python Software Foundation, Wilmington, DE, USA).

## Results

### Patients and study characteristics

Our analysis included 5990 patients from 65 sites across the UK, representing 1672 patients from 36 district general hospitals and 4318 patients from 29 tertiary referral centres. The geographic distribution of participants within our dataset has been published previously.^16^ The characteristics of individuals included in the analysis are shown in Table 1. Our population was broadly representative of patients undergoing high-risk surgery in the UK.^18^ Out of a total of 5990 patients, 539 (9.6%) had their surgery cancelled. There were no clear demographic differences (age, sex, ethnicity) between patients whose surgery proceeded and was cancelled. Patients with malignancy represented a smaller proportion of those that had their surgery cancelled than those whose surgery went ahead (36.4% vs 50.1%). Conversely, the prevalence of systemic comorbidities such as cardiac, pulmonary and neurological disease were relatively higher among those that experienced cancellation. The proportion of cases represented by each surgical speciality within our cancellation and proceeded cohorts were similar, illustrating differing caseloads but stable proportions across the two groups.

**Table 1.** A comparison of the demographics, comorbidities and primary surgical specialities of patients whose surgery did and did not proceed.

Patient characteristics	Overall *n* = 5990	Proceeded *n* = 5451	Cancelled *n* = 539
Median age	67 (56–76)	67 (56–76)	66 (55–76)
Male	3337 (55.7%)	3030 (55.6%)	307 (57.0%)
White ethnicity	4627 (77.2%)	4215 (77.3%)	412 (76.4%)
Comorbidities			
Chronic cardiac disease	1773 (29.6%)	1561 (28.6%)	212 (39.3%)
Chronic kidney disease	799 (13.3%)	710 (13.0%)	89 (16.5%)
Chronic liver disease	378 (6.3%)	336 (6.2%)	42 (7.8%)
Chronic neurological disease	662 (11.1%)	580 (10.6%)	82 (15.2%)
Chronic pulmonary disease	1477 (24.7%)	1314 (24.1%)	163 (30.2%)
Dementia	87 (1.5%)	77 (1.4%)	10 (1.9%)
Diabetes	1259 (21.0%)	1123 (20.6%)	136 (25.2%)
Hypertension	2789 (46.6%)	2522 (46.3%)	267 (49.5%)
Malignancy	2926 (48.9%)	2730 (50.1%)	196 (36.4%)
Metastasis	630 (10.5%)	586 (10.8%)	44 (8.2%)
Peripheral vascular disease	563 (9.4%)	503 (9.2%)	60 (11.1%)
Rheumatological disorders	463 (7.7%)	429 (7.9%)	34 (6.3%)
No significant comorbidities	649 (10.8%)	594 (10.9%)	55 (10.2%)
Surgical specialty performing procedure			
General surgery	2112 (35.3%)	1949 (35.8%)	163 (30.2%)
Gynaecological surgery	319 (5.3%)	296 (5.4%)	23 (4.3%)
Hepatobiliary surgery	653 (10.9%)	593 (10.9%)	60 (11.1%)
Orthopaedic surgery	782 (13.1%)	691 (12.7%)	91 (16.9%)
Otorhinolaryngology, maxillofacial and head & neck surgery	379 (6.3%)	341 (6.2%)	38 (7.1%)
Plastic surgery	58 (1.0%)	56 (1.0%)	2 (0.4%)
Renal & urological surgery	582 (9.7%)	514 (9.4%)	68 (12.6%)
Vascular surgery	518 (8.7%)	462 (8.5%)	56 (10.4%)
Unspecified	587 (9.8%)	549 (10.1%)	38 (7.1%)

Values are median (IQR) for age and otherwise counts and percentages.

### Cancellation

The reasons for cancellation of surgery are shown in Table 2. The most common reason for surgical cancellation was a lack of resources (51.0%), primarily bed shortages (37.1%). Where staff unavailability was the cause of cancellation (5.8%), personnel involved were surgeons (16), scrub staff (7), anaesthetists (4), operating department practitioners (2), interpreters (1) and interventional radiology staff (1). Inadequate optimisation (11.7%) and acute illness (10.9%) represented the majority of cancellations due to clinical reasons. Of those not optimised for surgery, specific reasons for cancellation included poor glucose control (2); uncontrolled hypertension (2); new cardiac issues identified on admission (2); and a low preoperative haemoglobin (1). The remainder (31) were cancelled following re-evaluation of the risks of surgery in reference to activity status and prior comorbidities. Administrative reasons for cancellation of surgery were not common (1.3%) and were mainly attributable to human errors such as booking the wrong date of surgery or forgetting to remove patients from the list if their surgery was rescheduled.

**Table 2.** Individuals whose surgeries did not proceed are grouped according to reason for cancellation.

Cancellation reasons	Count (%)
Resources	275 (51.0)
Lack of beds	201 (37.6)
Patients referred to level 1 (*n* = 3146)	73 (13.5)
Patients referred to level 2 (*n* = 1660)	72 (13.4)
Patients referred to level 3 (*n* = 1184)	56 (10.4)
Insufficient operating theatre capacity	34 (6.3)
Staff unavailable	31 (5.8)
Equipment problem	7 (1.3)
Blood products unavailable	2 (0.4)
Clinical reasons	153 (28.4)
Patient not optimised for surgery	63 (11.7)
Not medically optimised	38 (7.1)
Further investigations required	15 (2.8)
Preoperative instructions not followed	10 (1.9)
Patient acutely unwell	59 (10.9)
Change in surgical indication	28 (5.2)
Alternative therapy deemed more appropriate	7 (1.3)
Improvement of disease—surgery no longer indicated	7 (1.3)
Progression of disease—surgery no longer indicated	5 (0.9)
Unspecified change of indication	9 (1.7)
Required transfer to specialist centre	3 (0.6)
Personal reasons	26 (4.8)
Declined surgery	18 (3.3)
Did not attend	8 (1.5)
Administrative reasons	7 (1.3)
Unclear or not documented	78 (14.5)

Reasons are classified as resource related, clinical, personal and administrative. Cases are further subdivided by specific reasoning. Values are counts and percentages, with all percentages representing the proportion of those cancelled for each reason out of all 539 individuals whose surgery did not proceed.

The reasons for cancellation are stratified according to demographics, comorbidities and surgical speciality in Supplemental Table A1. Patients whose surgeries were cancelled for clinical reasons were older (68 years, IQR 58–78) than those whose surgeries were cancelled due to lack of resources (65 years, IQR 51–75). Patients with malignancy and cardiac disease, were overrepresented among those cancelled for clinical reasons.

### Changes in planned post-operative care destination

The rates of escalation and de-escalation of planned level of care and clinical outcomes in these subgroups, are shown in Table 3. Among the 5451 patients who proceeded with surgery, 81.3% received the anticipated level of care postoperatively, 3.9% required escalation and 14.8% were de-escalated to a lower level of care. Most of the escalations were from level 1 to level 2 (2.2%), while most de-escalations were from level 1 to 0 (5.6%). Death occurred in 204 cases (3.7%). Those that survived their admission exhibited a median duration of hospital stay of 6 days (IQR: 3–11). Progressive increases in mortality and duration of hospital stay were associated with admission to higher levels of care among those that received the planned level of care postoperatively. Patients escalated to a higher level of care had a higher mortality (6.2%) and stayed in hospital longer (10 days (IQR: 6–17)) than the overall cohort, whereas de-escalations demonstrated both a lower mortality (2.5%) and a shorter duration of hospital stay (4 days (IQR: 2–7)). For both escalations and de-escalations, outcomes worsened progressively as the gap between anticipated and actual level of care increased.

**Table 3.** Individuals whose surgeries proceeded are grouped according to whether they received the planned level of care postoperatively.

Outcome of care episode	Count (%)	Died (%)	Duration of hospital stay (IQR)
Surgery went ahead	5451	204 (3.7)	6 (3–11)
Received planned level of care	4432 (81.3)	171 (3.9)	6 (3–11)
Level 1	2457 (45.1)	67 (2.7)	5 (3–10)
Level 2	1138 (20.9)	49 (4.3)	8 (4–14)
Level 3	837 (15.4)	55 (6.6)	9 (5–16)
Escalated to a higher level of care	210 (3.9)	13 (6.2)	10 (6–17)
Level 1 to Level 2	119 (2.2)	4 (3.4)	7 (4–13)
Level 1 to Level 3	34 (0.6)	4 (11.8)	14 (8–27)
Level 2 to Level 3	57 (1.0)	5 (8.8)	14 (7–29)
De-escalated to a lower level of care	809 (14.8)	20 (2.5)	4 (2–7)
Level 1 to Level 0	303 (5.6)	12 (4.0)	3 (2–6)
Level 2 to Level 0	174 (3.2)	1 (0.6)	3 (1–6)
Level 3 to Level 0	141 (2.6)	0 (0.0)	4 (2–7)
Level 2 to Level 1	143 (2.6)	1 (0.7)	4 (2–7)
Level 3 to Level 1	13 (0.2)	1 (7.7)	7 (5–12)
Level 3 to Level 2	35 (0.6)	5 (14.3)	11 (5–16)

Cases are further subdivided by the anticipated and actual level of care received. The number of cases in each subgroup is displayed as a count and a percentage of the total cases that went ahead. The number of patients that died during their admission for each subgroup is also provided as a count, with the percentage representing the rate of mortality within that subgroup. Duration of hospital stay is presented as a median (IQR).

#### Escalation of care

Reasons for escalation to higher levels of care are shown in Table 4. The majority of patients were escalated for clinical reasons (53.3%) with substantially fewer re-allocated due to bed shortages (13.8%). Of those escalated for clinical reasons, most experienced deterioration postoperatively (29.5%) rather than intraoperatively (10.5%). Medical complications included cardiovascular instability, often requiring vasoactive infusions (26); desaturation and ventilatory issues (20); unanticipated need for dialysis (3); airway concerns (3); and cardiac arrest (2). Surgical complications included major haemorrhage (4); perforated viscera (2); and anastomotic leak (1). Where the clinical reasoning was not specified (13.3%), there were references to clinical assessments made on the day (4); significant medical comorbidities (2); and mortality scores (2). All escalations due to administrative errors (3) occurred because the incorrect level of care was booked preoperatively. The trends seen across all escalations were replicated within each subgroup when stratified according to the anticipated and actual level of care although, notably, no patients were escalated from level 1 to level 3 due to bed shortages.

**Table 4.** Individuals who received postoperative care in a higher level area than anticipated are grouped according to reason for escalation.

Escalation reasons	All escalations *n* = 210	Level 1 to Level 2 *n* = 119	Level 1 to Level 3 *n* = 34	Level 2 to Level 3 *n* = 57
Resources	29 (13.8%)	13 (10.9%)	0 (0.0%)	16 (28.1%)
Lack of beds	29 (13.8%)	13 (10.9%)	0 (0.0%)	16 (28.1%)
Clinical reasons	112 (53.3%)	56 (47.1%)	25 (73.5%)	31 (54.4%)
Postoperative deterioration	62 (29.5%)	27 (22.7%)	13 (38.2%)	22 (38.6%)
Medical complication	58 (27.6%)	26 (21.8%)	10 (29.4%)	22 (38.6%)
Surgical complication	4 (1.9%)	1 (0.8%)	3 (8.8%)	0 (0.0%)
Intraoperative deterioration	22 (10.5%)	9 (7.6%)	6 (17.6%)	7 (12.3%)
Medical complication	10 (4.8%)	3 (2.5%)	3 (8.8%)	4 (7.0%)
Surgical complication	9 (4.3%)	5 (4.2%)	2 (5.9%)	2 (3.5%)
Increased surgical complexity	3 (1.4%)	1 (0.8%)	1 (2.9%)	1 (1.8%)
Unspecified clinical decision	28 (13.3%)	20 (16.8%)	6 (17.6%)	2 (3.5%)
Administrative reasons	3 (1.4%)	3 (2.5%)	0 (0.0%)	0 (0.0%)
Unclear or not documented	66 (31.4%)	47 (39.5%)	9 (26.5%)	10 (17.5%)

Reasons are classified as resource related, clinical, personal and administrative. Cases are further subdivided by specific reasoning. Values are counts and percentages, with percentages representing the proportion within each column.

#### De-escalation of care

Reasons for de-escalation to lower levels of care are shown in Table 5. Similar to escalation, the most frequently cited reasons for de-escalation were clinical (61.2%). A common mechanism through which patients were de-escalated was following a postoperative review by the anaesthetist or intensivist in recovery (21.8%). Only a small minority of de-escalations were caused by a lack of beds, of which nearly all (42 of 49) were re-allocated from level 1 to level 0.

**Table 5.** Individuals who received postoperative care in a lower level area than anticipated are grouped according to reason for de-escalation.

De-escalation reasons	All de-escalations *n* = 809	Level 1 to Level 0 *n* = 303	Level 2 to Level 0 *n* = 174	Level 3 to Level 0 *n* = 141	Level 2 to Level 1 *n* = 143	Level 3 to Level 1 *n* = 13	Level 3 to Level 2 *n* = 35
Resources	49 (6.1%)	42 (13.9%)	3 (1.7%)	0 (0.0%)	4 (2.8%)	0 (0.0%)	0 (0.0%)
Lack of beds	49 (6.1%)	42 (13.9%)	3 (1.7%)	0 (0.0%)	4 (2.8%)	0 (0.0%)	0 (0.0%)
Clinical reasons	495 (61.2%)	156 (51.5%)	89 (51.1%)	113 (80.1%)	100 (69.9%)	11 (84.6%)	26 (74.3%)
De-escalated after postoperative assessment	176 (21.8%)	62 (20.5%)	32 (18.4%)	27 (19.1%)	46 (32.2%)	4 (30.8%)	5 (14.3%)
Lower surgical acuity	16 (2.0%)	8 (2.6%)	3 (1.7%)	2 (1.4%)	1 (0.7%)	2 (15.4%)	0 (0.0%)
Less extensive procedure performed	10 (1.2%)	6 (2.0%)	2 (1.1%)	1 (0.7%)	1 (0.7%)	0 (0.0%)	0 (0.0%)
Procedure abandoned	6 (0.7%)	2 (0.7%)	1 (0.6%)	1 (0.7%)	0 (0.0%)	2 (15.4%)	0 (0.0%)
Lower anaesthetic acuity	17 (2.1%)	5 (1.7%)	9 (5.2%)	3 (2.1%)	0 (0.0%)	0 (0.0%)	0 (0.0%)
Performed under regional anaesthesia	12 (1.5%)	4 (1.3%)	6 (3.4%)	2 (1.4%)	0 (0.0%)	0 (0.0%)	0 (0.0%)
Performed under local anaesthesia	4 (0.5%)	1 (0.3%)	2 (1.1%)	1 (0.7%)	0 (0.0%)	0 (0.0%)	0 (0.0%)
Performed under sedation	1 (0.1%)	0 (0.0%)	1 (0.6%)	0 (0.0%)	0 (0.0%)	0 (0.0%)	0 (0.0%)
Met criteria for day-case surgery	14 (1.7%)	5 (1.7%)	9 (5.2%)	0 (0.0%)	0 (0.0%)	0 (0.0%)	0 (0.0%)
Higher level bed booked as contingency	9 (1.1%)	7 (2.3%)	1 (0.6%)	0 (0.0%)	1 (0.7%)	0 (0.0%)	0 (0.0%)
Unspecified clinical decision	263 (32.5%)	69 (22.8%)	35 (20.1%)	81 (57.4%)	52 (36.4%)	5 (38.5%)	21 (60.0%)
Administrative reasons	2 (0.2%)	0 (0.0%)	1 (0.6%)	0 (0.0%)	1 (0.7%)	0 (0.0%)	0 (0.0%)
Unclear or not documented	263 (32.5%)	105 (34.7%)	81 (46.6%)	28 (19.9%)	38 (26.6%)	2 (15.4%)	9 (25.7%)

Reasons are classified as resource related, clinical, personal and administrative. Cases are further subdivided by specific reasoning. Values are counts and percentages, with percentages representing the proportion within each column.

When compared directly, patients that were escalated had a higher prevalence of significant comorbidities than those who were de-escalated (Supplemental Table B1). Escalations were more likely to be undergoing hepatobiliary, orthopaedic and vascular surgery, whereas general or urological surgery was more common among de-escalations.

## Discussion

### Main findings

This national multicentre analysis provides a contemporary overview of the perioperative landscape in the UK, focussing on pressures within enhanced and critical care pathways. Three main findings emerged. First, roughly one in 10 scheduled operations in this high-risk cohort were cancelled. Second, over half of cancellations were attributable to system-level resource constraints rather than clinical or patient factors. Third, divergence between planned and actual postoperative destination occurred in approximately one in five patients, with de-escalation being more common than escalation.

### Cancellation of surgery

The observed cancellation rate in this cohort was similar to national estimates reported for the UK general surgical population of over the same period.^3,19^ The predominance of resource-driven cancellations seen in this and previous analyses suggests that system capacity has become a critical bottleneck in perioperative care in the UK and beyond.^1,20^ Data from the wider surgical population demonstrated lower bed-related cancellation rates,^2,19^ illustrating a particular vulnerability among patients referred to higher levels of care to resource constraints.

Among clinically indicated cancellations, a substantial proportion were potentially avoidable. Cancellations due to inadequate optimisation or outstanding investigations and changes in surgical indication raise concerns about the effectiveness and responsiveness of current perioperative pathways. Long waiting times may allow interval clinical deterioration that static pre-assessment processes fail to detect.^21^ These findings highlight the value of dynamic digital perioperative systems capable of monitoring evolving patient risk, at the primary-secondary care or secondary-secondary care interfaces.^22^

The relatively higher proportion of patients with significant comorbidities among those that had their surgery cancelled compared to those whose surgery went ahead reflects the non-negotiable need for higher-acuity beds in certain contexts. Patients with cardiac and pulmonary diseases often require enhanced monitoring, thus when a bed is unavailable, surgery cannot safely proceed. This phenomenon is similarly reflected by the predominance of older patients with significant comorbidities among those whose surgeries were cancelled for clinical reasons, illustrating the pre-optimisation challenges associated with greater frailty.^23^ On the other hand, the relative underrepresentation of patients with malignancy among those whose surgery was cancelled suggests prioritisation of cases for which time to therapy has a well-established link with mortality.

### Escalation and de-escalation of planned post-operative level of care

Most patients received the anticipated level of care postoperatively. When divergence occurred, de-escalation was more common than escalation, typically reflecting more favourable intraoperative courses or reassessment following a postoperative clinical review. Compared to the cohort average, both mortality and duration of hospital stay were lower among those de-escalated to lower levels of care. While this may represent effective risk management, this apparent success should be interpreted cautiously. Although mortality and duration of hospital stay might be regarded as reasonable surrogates for population morbidity, this dataset did not capture the postoperative period in sufficient detail to encompass all clinically meaningful harm that might be accrued by individuals re-allocated to a lower level of care.^11,12,24^ Therefore, despite encouraging outcome data, the safety implications of de-escalation cannot be determined from this analysis. It is also worth noting that the mortality rate of those de-escalated from level 1 to level 0 falls within the range (1%–5%) that guidance frames as the optimal risk profile for provision of enhanced care. It is possible that the outcomes in this group would have been improved by admission to enhanced care, which is ultimately the purpose of these pathways and this practice diminishes the positive impacts of enhanced care at an institutional level by excluding a proportion of those that are most likely to benefit.

In contrast to cancellation, which was primarily a consequence of resource scarcity, the most common reasons for a change in the anticipated postoperative level of care were clinical. Where a change in postoperative destination did occur as a result of bed shortages, this practice was more prevalent among those that were escalated than those de-escalated. Together these observations illustrate that caution is generally applied when beds are not available, with clinicians preferentially arranging care in higher-level areas or deferring surgery to a later date. This study did not include a concurrent measure of enhanced or critical care bed capacity meaning we were unable to determine the extent to which such pressures influence decision-making for escalation and de-escalation. This is especially important for patients who have no immediate critical care needs but may benefit from a period of enhanced observation.

Nearly all those de-escalated due to a lack of beds were moved from level 1 to level 0. This represents a calculated risk being taken by clinicians on the day and suggests that enhanced care may, in practice, act as an interface between the ward and critical care where staffing ratios and the level of monitoring permit timely escalation or de-escalation of care to meet the clinical need. This dynamic use of beds within enhanced care may partly explain why cancellation is less common among patients referred to enhanced care rather than critical care.^16^

A recent service evaluation from South-West England identified similar reasons for de-escalation of post-operative destination.^25^ They concluded that the frequency with which patients were deemed ‘too well’ for higher levels of care reveals a learned tendency for how acutely unwell a patient needs to be to get admitted to critical care, a problem arising from historical under-provision of beds in these areas in the NHS. We similarly demonstrate that, in practice, many patients referred for higher level beds are de-escalated once intra- and postoperative factors are considered and add that this decision does not reflect in the population mortality or length of stay. It may well be the case that a substantial proportion of these individuals were initially over-triaged, perhaps as a result of institutional culture. However, this may also represent the inherent limitations of clinical judgement and risk scores, which in perioperative pathways are limited to the use of preoperative information, in the process of outcome prediction to identify those that will benefit most from planned critical care admission.^26–28^

### Clinical implications

Our findings emphasise the fragility of perioperative pathways reliant on limited enhanced and critical care capacity. Resource-driven cancellations represent both a patient burden and a major operational inefficiency, with cancelled theatre time estimated to cost the NHS hundreds of millions of pounds annually.^29^ Although the reasons for cancellation seemingly differ from those that cause changes in the level of care received postoperatively, these phenomena may be more accurately understood as part of a continuum of risk assessment. We show that the majority of escalations occur due to unavoidable clinical deterioration, whereas de-escalation often represents an act of prioritisation between resource use and an evolving risk profile, with both used as a mechanism to avoid cancellation.

We draw focus to the potential benefits of enhanced care facilities as an interface between the ward and critical care, in addition to their role in providing consistent and high-quality care that is tailored specifically to postoperative cohorts. For appropriately selected patients, providing enhanced monitoring for a pre-defined period after surgery may allow timely escalation and de-escalation in facilities with lower running costs than critical care.^13^ This might circumvent the harms of over-triage,^6^ while improving response times to deterioration among intermediate risk patients that would not have traditionally been admitted to critical care.^13^ The latter is of particular importance given the well-established link between delayed admission to critical care following postoperative deterioration and mortality.^30^ The practice of de-escalating patients following an immediate postoperative review does not consider that many of the complications that can lead to critical care admission, such as acute kidney injury, are rarely detected in the first few hours after surgery but may be more reliably identified over a 48-h period of enhanced monitoring.^31^ It remains uncertain whether enhanced care provides the proposed improvements in response times; whether they capture the population that is most likely to benefit; and whether their introduction has a favourable impact on health economic outcomes. Given that enhanced care is frequently used as an alternative to critical care due to substantial overlap between their populations,^32^ and whilst uncertainty remains regarding the benefits of perioperative critical care admission,^4,8–10^ there exists sufficient equipoise to compare the clinical and health economic outcomes of these two modalities in a randomised trial.

### Limitations

Our analysis is limited by the prevalence of missing data, 14.5% for cancellation and over 30% for changes in postoperative destination, preventing full interrogation of some subgroups. Our retrospective study design prevented formal scrutiny of the clinical decision-making in each scenario. At the interfaces between local investigators and clinical documentation and between the study authors and the free-text data submitted by these investigators, there was a risk of misinterpretation of the intent or meaning that a prospective design might have overcome. For example, decisions made because of a lack of resources may also have been justified on clinical grounds (e.g. de-escalating a patient based on a post-operative assessment that was requested due to a lack of higher-level bed) and the classification would depend on which was documented. Patterns in local reporting were also noted, where for example non-specific clinical reasons were given as reasoning for most patients, preventing capture of meaningful findings from those sites. Some specific causes of cancellation and re-allocation generated small subgroup numbers which were absorbed into larger categories for ease of interpretation. Larger numbers may therefore have provided greater granularity and led to more nuanced interpretations of these phenomena. Ethnicity data should be interpreted with caution as the quality of data collection for this was poor. For that reason, we broadly classified Ethnicity into ‘White’ and ‘Other’ which is in itself a bias. As this cohort includes only patients scheduled for surgery and referred to enhanced or critical care, no inferences can be made on appropriateness of referral or access disparities in earlier stages of the surgical pathway. Furthermore, as we did not study facilities accepting medical admissions exclusively, our findings cannot be extrapolated to medical enhanced care units. Finally, although study recruitment was UK-wide, centres providing data for this analysis were exclusively situated in England and Wales, with the majority of patients from South and West Yorkshire; Greater Manchester; Glamorgan; Oxfordshire; Cambridgeshire; and Greater London. This may further limit generalisability.

### Conclusion

In summary, among high-risk surgical patients with planned postoperative enhanced or critical care admission in the UK, almost one in 10 operations were cancelled and one in five experienced a deviation from their planned level of care. Resource constraints, in particular lack of beds, play an important role in cancellation and, where they do lead to a change in postoperative destination, this most commonly occurs between enhanced care and the ward. These findings reflect the risk–benefit decisions clinicians must make when balancing cancellation against proceeding without the anticipated level of support postoperatively. Clinically driven cancellations due to inadequate optimisation or interval changes in patient condition suggest that current perioperative pathways may not respond sufficiently to evolving risk. More integrated and dynamic perioperative systems may mitigate avoidable cancellations although prospective evaluation is required. While missing data limits interpretation of some findings, this large and broadly representative cohort provides contemporary insight into postoperative care planning in similar healthcare systems. Future research should seek to address longitudinal patient outcomes following same-day escalation and de-escalation of care and assess the clinical and health economic impacts of increasing reliance on enhanced care as an alternative to critical care.

## Supplemental Material

sj-docx-1-inc-10.1177_17511437261495043 – Supplemental material for Surgical cancellation and changes in postoperative level of care among high and moderate risk surgical patients in the UK Supplemental material, sj-docx-1-inc-10.1177_17511437261495043 for Surgical cancellation and changes in postoperative level of care among high and moderate risk surgical patients in the UK by Joanna K. L. Wong, Rosanna Sasson and Christopher Oddy in Journal of the Intensive Care Society

sj-docx-2-inc-10.1177_17511437261495043 – Supplemental material for Surgical cancellation and changes in postoperative level of care among high and moderate risk surgical patients in the UK Supplemental material, sj-docx-2-inc-10.1177_17511437261495043 for Surgical cancellation and changes in postoperative level of care among high and moderate risk surgical patients in the UK by Joanna K. L. Wong, Rosanna Sasson and Christopher Oddy in Journal of the Intensive Care Society

sj-docx-3-inc-10.1177_17511437261495043 – Supplemental material for Surgical cancellation and changes in postoperative level of care among high and moderate risk surgical patients in the UK Supplemental material, sj-docx-3-inc-10.1177_17511437261495043 for Surgical cancellation and changes in postoperative level of care among high and moderate risk surgical patients in the UK by Joanna K. L. Wong, Rosanna Sasson and Christopher Oddy in Journal of the Intensive Care Society

sj-docx-4-inc-10.1177_17511437261495043 – Supplemental material for Surgical cancellation and changes in postoperative level of care among high and moderate risk surgical patients in the UK Supplemental material, sj-docx-4-inc-10.1177_17511437261495043 for Surgical cancellation and changes in postoperative level of care among high and moderate risk surgical patients in the UK by Joanna K. L. Wong, Rosanna Sasson and Christopher Oddy in Journal of the Intensive Care Society

sj-docx-6-inc-10.1177_17511437261495043 – Supplemental material for Surgical cancellation and changes in postoperative level of care among high and moderate risk surgical patients in the UK Supplemental material, sj-docx-6-inc-10.1177_17511437261495043 for Surgical cancellation and changes in postoperative level of care among high and moderate risk surgical patients in the UK by Joanna K. L. Wong, Rosanna Sasson and Christopher Oddy in Journal of the Intensive Care Society

sj-docx-7-inc-10.1177_17511437261495043 – Supplemental material for Surgical cancellation and changes in postoperative level of care among high and moderate risk surgical patients in the UK Supplemental material, sj-docx-7-inc-10.1177_17511437261495043 for Surgical cancellation and changes in postoperative level of care among high and moderate risk surgical patients in the UK by Joanna K. L. Wong, Rosanna Sasson and Christopher Oddy in Journal of the Intensive Care Society

sj-docx-8-inc-10.1177_17511437261495043 – Supplemental material for Surgical cancellation and changes in postoperative level of care among high and moderate risk surgical patients in the UK Supplemental material, sj-docx-8-inc-10.1177_17511437261495043 for Surgical cancellation and changes in postoperative level of care among high and moderate risk surgical patients in the UK by Joanna K. L. Wong, Rosanna Sasson and Christopher Oddy in Journal of the Intensive Care Society

sj-xlsx-5-inc-10.1177_17511437261495043 – Supplemental material for Surgical cancellation and changes in postoperative level of care among high and moderate risk surgical patients in the UK Supplemental material, sj-xlsx-5-inc-10.1177_17511437261495043 for Surgical cancellation and changes in postoperative level of care among high and moderate risk surgical patients in the UK by Joanna K. L. Wong, Rosanna Sasson and Christopher Oddy in Journal of the Intensive Care Society

## Data Availability

Our full dataset could not be made publicly available due to use of sensitive and confidential information. However, fully anonymised data and statistical code are available upon reasonable request through direct contact with the corresponding author.*
