# Supplementary material for: Surgical cancellation and changes in postoperative level of care among high and moderate risk surgical patients in the UK
Source: J Intensive Care Soc. 2026 Oct 12:17511437261495043. Online ahead of print. doi: 10.1177/17511437261495043 (PMC13658631; doi:10.1177/17511437261495043)
Supplement: Supplementary file 1 [file sj-docx-1-inc-10.1177_17511437261495043.docx]

**Appendix A**	The REPACC Study Investigators ordered by hospital and role.

| Hospital | Name | Role |
| --- | --- | --- |
| Ashford and St. Peter's Hospital NHS Foundation Trust | Mohamed Hussein | Trainee Lead |
| Ashford and St. Peter's Hospital NHS Foundation Trust | Kazi Farhana Akhter | Trainee Investigator |
| Ashford and St. Peter's Hospital NHS Foundation Trust | Konye Anthonia Ochonogor | Trainee Investigator |
| Ashford and St. Peter's Hospital NHS Foundation Trust | Tanya Elizabeth Spencer | Trainee Investigator |
| Ashford and St. Peter's Hospital NHS Foundation Trust | Nicky Carroll | Consultant Supervisor |
| Ashford and St. Peter's Hospital NHS Foundation Trust | Zain Malik | Consultant Supervisor |
| Ashford and St. Peter's Hospital NHS Foundation Trust | Anja Sabine Daniela Kuttler | Consultant Supervisor |
| Barnet Hospital | Madeleine Gray | Trainee Lead |
| Barnet Hospital | Cathy Zhang | Trainee Investigator |
| Barnet Hospital | Marie Smith | Trainee Investigator |
| Barnet Hospital | Snehal Kale | Trainee Investigator |
| Barnet Hospital | Sumeet Gajbhar | Trainee Investigator |
| Barnet Hospital | Nathalie Stevenson | Consultant Supervisor |
| Barnsley Hospital | Jennifer Mullan | Trainee Lead |
| Barnsley Hospital | Joshua Hulman | Trainee Investigator |
| Barnsley Hospital | Ken Inweregbu | Consultant Supervisor |
| Broomfield Hospital | Juan Michael Jansen van Vuuren | Trainee Lead |
| Broomfield Hospital | Jatinder Stanley | Trainee Investigator |
| Broomfield Hospital | Arun Sahni | Consultant Supervisor |
| Cambridge University Hospitals | Caroline Phillips | Trainee Lead |
| Cambridge University Hospitals | Lara Allen | Trainee Lead |
| Cambridge University Hospitals | Jessica Hickman | Trainee Investigator |
| Cambridge University Hospitals | Georgina Singleton | Consultant Supervisor |
| Cambridge University Hospitals | Lisa Grimes | Consultant Supervisor |
| Castle Hill Hospital | Simon Chung Yan Lam | Trainee Lead |
| Castle Hill Hospital | Dinesh Tumu | Trainee Investigator |
| Castle Hill Hospital | Gayatri Khanvelkar | Trainee Investigator |
| Castle Hill Hospital | Rohit Patil | Trainee Investigator |
| Castle Hill Hospital | Prasad Lanka | Consultant Supervisor |
| Central Middlesex Hospital | James Womersley | Trainee Lead |
| Central Middlesex Hospital | Emma Smith | Trainee Investigator |
| Charing Cross Hospital | Helen Köwitz Arroyo | Trainee Lead |
| Charing Cross Hospital | Jahnavi Priya | Trainee Lead |
| Charing Cross Hospital | Dafydd Lloyd | Consultant Supervisor |
| Chelsea and Westminster Hospital | Ceit Cunningham | Trainee Lead |
| Chelsea and Westminster Hospital | Richard Snooks | Trainee Lead |
| Chelsea and Westminster Hospital | Aimi Lara Emilie Jeanes | Trainee Investigator |
| Chelsea and Westminster Hospital | Lara Jeanes | Trainee Investigator |
| Chelsea and Westminster Hospital | Tamsyn Glover | Trainee Investigator |
| Chelsea and Westminster Hospital | Roger Davies | Consultant Supervisor |
| City and Sandwell Hospitals Birmingham | Emily J Croft | Trainee Lead |
| City and Sandwell Hospitals Birmingham | Nipun Agarwal | Consultant Supervisor |
| Core Committee | Amanda Davis | Core Committee |
| Core Committee | Henry Lewith | Core Committee |
| Core Committee | Paolo Perella | Core Committee |
| Core Committee | Roshni Molls | Core Committee |
| Core Committee | Thomas Davies | Core Committee |
| Core Committee | Danny J N Wong | Core Committee |
| Croydon University Hospital | Thomas Craven | Trainee Lead |
| Croydon University Hospital | Barnaby Dykes | Trainee Investigator |
| Croydon University Hospital | Gianfranco Messina | Trainee Investigator |
| Croydon University Hospital | Islam Abousharkh | Trainee Investigator |
| Croydon University Hospital | James Brown | Trainee Investigator |
| Croydon University Hospital | Sarah Kipling | Trainee Investigator |
| Croydon University Hospital | Sodha Meera | Trainee Investigator |
| Croydon University Hospital | Stephanie Davis | Trainee Investigator |
| Croydon University Hospital | Maria Chereshneva | Consultant Supervisor |
| Croydon University Hospital | Sundar Raj Ashok | Consultant Supervisor |
| Derby Hospital | Craig Morris | Consultant Supervisor |
| Ealing Hospital | Thomas Bacarese-Hamilton | Trainee Lead |
| Ealing Hospital | Jack Gallagher | Trainee Investigator |
| Ealing Hospital | Nicola K Pearson | Consultant Supervisor |
| Frimley Hospital | Katarzyna Anna Marasinska | Trainee Lead |
| Frimley Hospital | William Roberts | Trainee Lead |
| Frimley Hospital | Vikram Singh | Trainee Investigator |
| Frimley Hospital | Benjamin Ledsom | Trainee Investigator |
| Frimley Hospital | Bhavna Bhalla | Trainee Investigator |
| Frimley Hospital | Sioned Phillips | Consultant Supervisor |
| Guy's and St Thomas NHS Foundation Trust | David Bailey | Trainee Lead |
| Guy's and St Thomas NHS Foundation Trust | Jacob Lawson | Trainee Lead |
| Guy's and St Thomas NHS Foundation Trust | Adriano Ferrante | Trainee Investigator |
| Guy's and St Thomas NHS Foundation Trust | Aldo Alberto Bignamini | Trainee Investigator |
| Guy's and St Thomas' NHS Foundation Trust | Adam Beebeejaun | Trainee Investigator |
| Guy's and St Thomas' NHS Foundation Trust | David Bailey | Trainee Investigator |
| Guy's and St Thomas' NHS Foundation Trust | Georgia Boniface | Trainee Investigator |
| Guy's and St Thomas' NHS Foundation Trust | Jen VanRoss | Trainee Investigator |
| Guy's and St Thomas' NHS Foundation Trust | Larissa Matveeva | Trainee Investigator |
| Guy's and St Thomas' NHS Foundation Trust | Sherpal Singh | Trainee Investigator |
| Guy's and St Thomas' NHS Foundation Trust | Soo Yoon | Trainee Investigator |
| Guy's and St Thomas' NHS Foundation Trust | Zhi Yap | Trainee Investigator |
| Hammersmith Hospital | Divya Gautam | Trainee Lead |
| Hammersmith Hospital | Omnia Askar | Trainee Lead |
| Hammersmith Hospital | Sanooj Soni | Consultant Supervisor |
| Hillingdon Hospital | Priyanka Tewari | Trainee Lead |
| Hillingdon Hospital | Ankita Deshpande | Trainee Investigator |
| Hillingdon Hospital | Kimberly Kok | Trainee Investigator |
| Hillingdon Hospital | Neil Gaurav Verma | Consultant Supervisor |
| Hillingdon Hospital | Nigel West | Consultant Supervisor |
| Hull Royal Infirmary | Tanumoy Maulick | Trainee Lead |
| Hull Royal Infirmary | John Robson | Trainee Investigator |
| Hull Royal Infirmary | Ikechukwu Igwebuike | Trainee Investigator |
| Hull Royal Infirmary | Balaji Rajamani | Consultant Supervisor |
| Kent and Canterbury Hospital | Fionnuala Durrant | Trainee Lead |
| Kent and Canterbury Hospital | John McDonald | Trainee Investigator |
| Kent and Canterbury Hospital | Jude Spedding | Trainee Investigator |
| Kent and Canterbury Hospital | Leo Worley | Trainee Investigator |
| Kent and Canterbury Hospital | Ritoo Kapoor | Consultant Supervisor |
| King George Hospital | Ivana Kaljevic | Trainee Lead |
| Kings College Hospital | Sarah Brown | Trainee Lead |
| Kings College Hospital | Benedicte Sjoflot | Trainee Investigator |
| Kings College Hospital | Sanya Patel | Trainee Investigator |
| Kings College Hospital | Akshay Pradhan | Trainee Investigator |
| Kings College Hospital | Arif Ahmed | Trainee Investigator |
| Kings College Hospital | Ee Lyn Chan | Trainee Investigator |
| Kings College Hospital | Jenny Olsson | Trainee Investigator |
| Kings College Hospital | Ravi Bhatia | Consultant Supervisor |
| Kings Mill Hospital | Marie Harrison | Trainee Lead |
| Kings Mill Hospital | Michael Burnley | Trainee Investigator |
| Kings Mill Hospital | Deepak Subramani | Consultant Supervisor |
| Leeds Teaching Hospitals Trust | Rachel Holmes | Trainee Lead |
| Leeds Teaching Hospitals Trust | Moh’d Adnan Hasan Yousef | Trainee Lead |
| Leeds Teaching Hospitals Trust | Lucy Marshall | Trainee Investigator |
| Leeds Teaching Hospitals Trust | Rebecca King | Trainee Investigator |
| Leeds Teaching Hospitals Trust | William Flinn | Trainee Investigator |
| Leeds Teaching Hospitals Trust | Ayman Salmona | Trainee Investigator |
| Leeds Teaching Hospitals Trust | Hisham Mohamed Riad | Trainee Investigator |
| Leeds Teaching Hospitals Trust | Prajith Sanjeewa Amarasekara | Trainee Investigator |
| Leeds Teaching Hospitals Trust | Ruth Charlesworth | Trainee Investigator |
| Leeds Teaching Hospitals Trust | Valeria Ortiz Martinez | Trainee Investigator |
| Leeds Teaching Hospitals Trust | Zahid Furqan | Trainee Investigator |
| Leeds Teaching Hospitals Trust | Alix Gerard | Nursing Lead |
| Leeds Teaching Hospitals Trust | Louise White | Nursing Lead |
| Leeds Teaching Hospitals Trust | Catherine Moriarty | Nursing Lead |
| Leeds Teaching Hospitals Trust | Caroline Thomas | Consultant Supervisor |
| Lincoln County Hospital | Benjamin Kidner | Trainee Lead |
| Lincoln County Hospital | Benjamin Kidner | Trainee Lead |
| Lincoln County Hospital | Rebecca Holyland | Trainee Investigator |
| Lincoln County Hospital | Varun Aggarwal | Consultant Supervisor |
| Lister Hospital | Talia Wieder | Trainee Lead |
| Lister Hospital | Gursimren Lidder | Trainee Investigator |
| Lister Hospital | Robert Connolly | Trainee Investigator |
| Lister Hospital | Pietro Ferranti | Consultant Supervisor |
| Maidstone and Tunbridge Wells NHS Trust | Camila Maria de Oliveira | Trainee Lead |
| Maidstone and Tunbridge Wells NHS Trust | Manju Sharma | Trainee Lead |
| Maidstone and Tunbridge Wells NHS Trust | Meithem Ali | Trainee Investigator |
| Maidstone and Tunbridge Wells NHS Trust | Amy Ackerley | Nursing Lead |
| Maidstone and Tunbridge Wells NHS Trust | Rebecca Seaman | Nursing Lead |
| Maidstone and Tunbridge Wells NHS Trust | Kate Stannard | Consultant Supervisor |
| Maidstone and Tunbridge Wells NHS Trust | James Wood | Consultant Supervisor |
| Manchester Royal Infirmary | Eslam Elsayed | Trainee Lead |
| Manchester Royal Infirmary | Arun Prasad Ravichandran | Trainee Investigator |
| Manchester Royal Infirmary | Prerna Shaktawat | Trainee Investigator |
| Manchester Royal Infirmary | Rashid Hussain | Trainee Investigator |
| Manchester Royal Infirmary | Samyuktha Raj | Trainee Investigator |
| Manchester Royal Infirmary | Yousuf Abusamra | Trainee Investigator |
| Manchester Royal Infirmary | Gareth Kitchen | Consultant Supervisor |
| Mid and South Essex NHS Foundation Trust | Alexander Fowler | Statistical Consultant |
| Mid Yorkshire Teaching NHS Trust | Ameila Milton | Trainee Lead |
| Mid Yorkshire Teaching NHS Trust | Chloe Start | Trainee Lead |
| Mid Yorkshire Teaching NHS Trust | Adam Mills | Trainee Investigator |
| Mid Yorkshire Teaching NHS Trust | Benjamin Hofland-Ward | Trainee Investigator |
| Mid Yorkshire Teaching NHS Trust | George Humble | Trainee Investigator |
| Mid Yorkshire Teaching NHS Trust | Kate Preston | Trainee Investigator |
| Mid Yorkshire Teaching NHS Trust | Matthew Smith | Trainee Investigator |
| Mid Yorkshire Teaching NHS Trust | Milena Vannahme | Trainee Investigator |
| Mid Yorkshire Teaching NHS Trust | Brendan Sloan | Consultant Supervisor |
| Morriston Hospital | Catherine Chapman | Trainee Lead |
| Morriston Hospital | Helen Ivatt | Trainee Investigator |
| Morriston Hospital | Steve Davies | Trainee Investigator |
| Morriston Hospital | Tristan Edey | Trainee Investigator |
| Morriston Hospital | Nia Wyn Davies | Consultant Supervisor |
| National Hospital for Neurology and Neurosurgery | Justin Maini | Trainee Lead |
| National Hospital for Neurology and Neurosurgery | Thomas Hirst | Trainee Lead |
| National Hospital for Neurology and Neurosurgery | Dona Saha | Trainee Investigator |
| National Hospital for Neurology and Neurosurgery | Drew Harding | Trainee Investigator |
| National Hospital for Neurology and Neurosurgery | Gilbert Thomas-Black | Trainee Investigator |
| National Hospital for Neurology and Neurosurgery | Georgia Mathieson | Trainee Investigator |
| Newham University Hospital | Francis Elechi | Trainee Lead |
| Newham University Hospital | Kavita Patel | Trainee Investigator |
| Newham University Hospital | Maia Shivani Yashwini Back | Trainee Investigator |
| Newham University Hospital | Freddie Weyman | Trainee Investigator |
| Newham University Hospital | Ketan Kataria | Trainee Investigator |
| Newham University Hospital | Thomas Fowler | Trainee Investigator |
| Newham University Hospital | Bhavesh Gohil | Consultant Supervisor |
| North Middlesex University Hospital | Rohit Hanmanta Bhandari | Trainee Lead |
| North Middlesex University Hospital | Sonali Gupta | Trainee Lead |
| North Middlesex University Hospital | AlexKumar Patel | Trainee Investigator |
| North Middlesex University Hospital | Kritika Rathour | Trainee Investigator |
| North Middlesex University Hospital | Rohit Rohit | Consultant Supervisor |
| Oxford University Hospitals NHS Foundation Trust | Estelle Maria D'Cunha | Trainee Lead |
| Oxford University Hospitals NHS Foundation Trust | Ammara Khan | Trainee Investigator |
| Oxford University Hospitals NHS Foundation Trust | Anjali Ashok Menon | Trainee Investigator |
| Oxford University Hospitals NHS Foundation Trust | Arun Thirunavukarasu | Trainee Investigator |
| Oxford University Hospitals NHS Foundation Trust | Mansi Gandhi | Trainee Investigator |
| Oxford University Hospitals NHS Foundation Trust | James Goetz | Trainee Investigator |
| Oxford University Hospitals NHS Foundation Trust | Maria Pikoula | Trainee Investigator |
| Oxford University Hospitals NHS Foundation Trust | Menaka Iyer | Trainee Investigator |
| Oxford University Hospitals NHS Foundation Trust | Sarah Hudson | Trainee Investigator |
| Oxford University Hospitals NHS Foundation Trust | Shweta Appiah | Trainee Investigator |
| Oxford University Hospitals NHS Foundation Trust | Sophie Ledsome | Trainee Investigator |
| Oxford University Hospitals NHS Foundation Trust | Kirstin Strathie | Trainee Investigator |
| Oxford University Hospitals NHS Foundation Trust | Tejal Kothari | Trainee Investigator |
| Oxford University Hospitals NHS Foundation Trust | Yashasvi Perera | Trainee Investigator |
| Oxford University Hospitals NHS Foundation Trust | James Day | Consultant Supervisor |
| Prince Charles Hospital | Alexander Johnson | Trainee Lead |
| Princess of Wales Hospital | Adam Phillips | Trainee Lead |
| Princess of Wales Hospital | Elin Rowlands | Trainee Investigator |
| Princess of Wales Hospital | Elizabeth Grant | Trainee Investigator |
| Princess of Wales Hospital | Jessica Phillips | Trainee Investigator |
| Princess Royal University Hospital | Anna Forbes | Trainee Lead |
| Princess Royal University Hospital | Thomas Reed | Trainee Lead |
| Princess Royal University Hospital | Joseph A Gallaher | Trainee Investigator |
| Princess Royal University Hospital | Rebecca Mitchell | Trainee Investigator |
| Princess Royal University Hospital | Hayley Kaye | Consultant Supervisor |
| Queen Elizabeth Queen Mother Hospital | Charlene Barter | Trainee Lead |
| Queen Elizabeth Queen Mother Hospital | Tamer Younes | Trainee Lead |
| Queen Elizabeth Queen Mother Hospital | Moustafa Zahra | Trainee Investigator |
| Queen Elizabeth Queen Mother Hospital | Sameer Qasala | Trainee Investigator |
| Queen Elizabeth Queen Mother Hospital | Sajith Damodaran | Consultant Supervisor |
| Queen's Hospital Romford | Hannah Nugent | Trainee Lead |
| Queen's Hospital Romford | Mandeep Phull | Consultant Supervisor |
| Regional Lead | Adam J Boulton | Regional Lead |
| Regional Lead | Adam Green | Regional Lead |
| Regional Lead | Ahmed Ibrahim | Regional Lead |
| Regional Lead | Brigitte Baxter | Regional Lead |
| Regional Lead | Mark Burnett | Regional Lead |
| Regional Lead | Dominic Lowcock | Regional Lead |
| Regional Lead | Orla Gillman | Regional Lead |
| Regional Lead | Sejal Modasia | Regional Lead |
| Regional Lead | Gareth Ifan James Davey | Regional Lead |
| Regional Lead | Graham Bolton | Regional Lead |
| Regional Lead | Karthik Iyer | Regional Lead |
| Regional Lead | Michael P Coulton | Regional Lead |
| Regional Lead | Olivia Bools | Regional Lead |
| Regional Lead | Paul Bramley | Regional Lead |
| Regional Lead | Sarah Towey | Regional Lead |
| Rotherham Hospital | Aaron Owen | Trainee Lead |
| Rotherham Hospital | Jonathan Emberey | Trainee Lead |
| Rotherham Hospital | Anil Hormis | Consultant Supervisor |
| Rotherham Hospital | Clare Windsor | Consultant Supervisor |
| Royal Albert Edward Infirmary | Rebecca Murphy | Trainee Lead |
| Royal Albert Edward Infirmary | Ana Orozco | Trainee Investigator |
| Royal Albert Edward Infirmary | Maxime Gibbons | Trainee Investigator |
| Royal Albert Edward Infirmary | Robin Helsby | Trainee Investigator |
| Royal Albert Edward Infirmary | Arun Mohanraj | Consultant Supervisor |
| Royal Blackburn Hospital | Alexandra Gray-Renfrew | Trainee Lead |
| Royal Blackburn Hospital | Alison Thorne | Trainee Investigator |
| Royal Blackburn Hospital | Elizabeth Whittaker | Trainee Investigator |
| Royal Blackburn Hospital | Jesymin Jia Min Choong | Trainee Investigator |
| Royal Blackburn Hospital | Vikram Nagarajan | Trainee Investigator |
| Royal Blackburn Hospital | Anuradha Kurvey | Consultant Supervisor |
| Royal Bolton Hospital | Claire Pollington | Trainee Lead |
| Royal Bolton Hospital | Saadia Shahzad | Trainee Investigator |
| Royal Bolton Hospital | Stephanie Alsop | Trainee Investigator |
| Royal Bolton Hospital | Madhu Balasubramaniam | Consultant Supervisor |
| Royal Free Hospital | Eleanor Shaw | Trainee Lead |
| Royal Free Hospital | Rachel Grant | Trainee Investigator |
| Royal Free Hospital | Alexandra Cockroft | Trainee Investigator |
| Royal Free Hospital | David Ivan Wells | Trainee Investigator |
| Royal Free Hospital | Isabel Greaves | Trainee Investigator |
| Royal Free Hospital | Oliver Eastwood | Trainee Investigator |
| Royal Free Hospital | Judith Cheong-Leen | Consultant Supervisor |
| Royal Preston Hospital | Thomas Capstick | Trainee Lead |
| Salford Royal Hospital | Urbi Sen | Trainee Investigator |
| Salford Royal Hospital | Leanne Darwin | Consultant Supervisor |
| Scarborough General Hospital | Uneesa Mahmood | Trainee Lead |
| Scarborough General Hospital | Alastair Savage | Trainee Investigator |
| Scarborough General Hospital | Adedolapo Adejumo | Trainee Investigator |
| Scarborough General Hospital | Sam Murgatroyd | Trainee Investigator |
| Scarborough General Hospital | John Mensah | Consultant Supervisor |
| Sheffield Teaching Hospitals | Victoria Lowden | Trainee Lead |
| Sheffield Teaching Hospitals | Alex Chatburn | Trainee Investigator |
| Sheffield Teaching Hospitals | Christopher Harrison | Trainee Investigator |
| Sheffield Teaching Hospitals | Erica Morris | Trainee Investigator |
| Sheffield Teaching Hospitals | Jake Winterbottom | Trainee Investigator |
| Sheffield Teaching Hospitals | Samuel Allen | Trainee Investigator |
| Sheffield Teaching Hospitals | Viren Kadodwala | Trainee Investigator |
| Sheffield Teaching Hospitals | Matthew Needham | Consultant Supervisor |
| Singleton Hospital | Matthew Holland | Trainee Lead |
| Singleton Hospital | Matthew Holland | Trainee Lead |
| Singleton Hospital | Eleanor Lewis | Consultant Supervisor |
| St George’s Hospital | Gareth Ifan James Davey | Trainee Lead |
| St George’s Hospital | Nicoletta Fossati | Consultant Supervisor |
| St George's Hospital | Hannah Gardiner | Trainee Investigator |
| St George's Hospital | Prateek Nalwaya | Trainee Investigator |
| St George's Hospital | Adyasha Das | Trainee Investigator |
| St George's Hospital | Duncan Baily | Trainee Investigator |
| St Mary's Hospital | Cindy Luximon | Trainee Lead |
| St Mary's Hospital | Alex Wickham | Consultant Supervisor |
| The Royal Orthopaedic Hospital | James Heggie | Trainee Lead |
| The Royal Orthopaedic Hospital | Aamer Mughal | Trainee Investigator |
| The Royal Orthopaedic Hospital | Daniel Greaves | Trainee Investigator |
| The Royal Orthopaedic Hospital | Narendra Siddaiah | Consultant Supervisor |
| The York Hospital | Gabriel Chee En Ngui | Trainee Lead |
| The York Hospital | Thomas Hawthorne | Trainee Lead |
| The York Hospital | Katie Wilson | Trainee Investigator |
| The York Hospital | Kirstie Wright | Trainee Investigator |
| The York Hospital | Nuruljannah Ismail | Trainee Investigator |
| The York Hospital | Tochukwu Bede Oluh | Trainee Investigator |
| The York Hospital | Murray Williams | Consultant Supervisor |
| University College Hospital | Ashli Antoine | Trainee Lead |
| University College Hospital | Peter M Odor | Consultant Supervisor |
| University Hospital Wales | Naomi Jordan | Trainee Lead |
| University Hospital Wales | Sarah Elgarf | Trainee Lead |
| University Hospital Wales | Gruffudd Thomas | Trainee Investigator |
| University Hospital Wales | Catherine Britton-Jones | Consultant Supervisor |
| Warwick Hospital | Bejal Patel | Trainee Lead |
| Warwick Hospital | Shona Sandhu | Trainee Investigator |
| Warwick Hospital | Ben Wooldridge | Consultant Supervisor |
| West Suffolk Hospital | Saarah Rana | Trainee Lead |
| West Suffolk Hospital | Zi Chao Soo | Trainee Lead |
| West Suffolk Hospital | Amitesh Chugh | Trainee investigator |
| West Suffolk Hospital | Danboy Ayodeji | Trainee investigator |
| West Suffolk Hospital | Andrew Tang | Consultant Supervisor |
| William Harvey Hospital | James P King | Trainee Lead |
| William Harvey Hospital | Diana Neely | Consultant Supervisor |
| Wrexham Maelor Hospital | Nithin Jayan | Trainee Lead |
| Wrexham Maelor Hospital | Najade Sheriff | Trainee Lead |
| Wrexham Maelor Hospital | Anna Williams | Consultant Supervisor |
| Ysbyty Glan Clwyd | Beatrice Meilak | Trainee Lead |
| Ysbyty Glan Clwyd | Joe Woodcock | Trainee Lead |
| Ysbyty Glan Clwyd | Morwenna Coultate | Trainee Investigator |
| Ysbyty Glan Clwyd | Venkat Sundaram | Consultant Supervisor |
