# Supplementary material for: Surgical cancellation and changes in postoperative level of care among high and moderate risk surgical patients in the UK
Source: J Intensive Care Soc. 2026 Oct 12:17511437261495043. Online ahead of print. doi: 10.1177/17511437261495043 (PMC13658631; doi:10.1177/17511437261495043)
Supplement: Supplementary file 3 [file sj-docx-3-inc-10.1177_17511437261495043.docx]

**Appendix C**

Below are illustrative screenshots of the patient data case report form. Please download the full file from the following link:

<https://docs.google.com/spreadsheets/d/11tR99di703l6DSO8JNxHCqDeUlTiu6vx/edit?usp=drive_link&ouid=101343698314211870070&rtpof=true&sd=true>
