# Supplementary material for: Surgical cancellation and changes in postoperative level of care among high and moderate risk surgical patients in the UK
Source: J Intensive Care Soc. 2026 Oct 12:17511437261495043. Online ahead of print. doi: 10.1177/17511437261495043 (PMC13658631; doi:10.1177/17511437261495043)
Supplement: Supplementary file 4 [file sj-docx-4-inc-10.1177_17511437261495043.docx]

# Site Setup Guide

# The structural and organisational impacts of perioperative enhanced care services in the UK: A Retrospective Evaluation of Post-operative Alternatives to Critical Care (REPACC)

Pan-London Perioperative Audit & Research Network

Protocol Date: 10/07/24

IRAS Project ID: 338772

Correspondence

Chief Investigator (CI):

Dr Christopher Oddy

Department of Anaesthesia

Kingston Hospital

Galsworthy Rd

Kingston upon Thames

KT2 7QB

Email: [repacc.plan@gmail.com](mailto:repacc.plan@gmail.com)

# How to get started

Thank you for your interest in our project! We have created a guide to help you throughout the data collection process. This guide is very detailed, but don’t worry, the data collection process is simple and usually doesn’t take very long!

The first stage of the process is identifying a team of enthusiastic trainees and a consultant supervisor. We recommend that each site has a trainee lead, and – depending on the size of your hospital – between 1 and 3 additional team members. The lead consultant should be able to assist with completing the survey component of the study. In particular, it may be useful to involve the perioperative lead at your site to supervise. It may be useful to give potential team members a sense for the time commitment required. The expected time commitments for data collection are as follows:

- Trainee lead – **3-4 full days** with some additional time spent emailing and coordinating.
- Consultant lead – **<1 full day**.
- Trainee investigators – **1-2 full days** of data collection.

The data collection flowchart on the following page summarises the process that follows. This starts with registering the project with your local research and development team. We will need to hear that you have had the project approved at your site. Our set-up checklist is below to get you off the ground!

- Identify trainee team members (2-4 recommended)
- Identify a consultant supervisor
- Obtain approvals from research & development team
- Email OID to [khft.researchgovernance@nhs.net](mailto:khft.researchgovernance@nhs.net)

# Data collection flowchart

# Step-by-step guide

**Step 1a**

## Registration with your research & development department

The REPACC study is sponsored by the Research & Innovation department at Kingston Hospital and has obtained both information governance and HRA approval for national rollout of the current protocol. To ensure adequate oversight, each participating centre must register the project with their local research and development department. The process will involve an independent assessment of the particular risks posed by undertaking data collection at each site. Approval by the local research and development department is mandatory before data collection can begin.

The research and development department will typically need:

- A summary of the type of data that will be collected.
- Information concerning how patient identifiable data will be accessed, stored, and distributed – and with whom this will be shared.
- A rough timescale for data collection.
- A full list of staff members affiliated with the project at your site.
- The IRAS project ID (338772).

All of this information is available in the study protocol (File: Study Protocol). It may be sufficient to submit this in its current format or, alternatively, your local research and development team may require you to submit a locally developed proforma. For this purpose we have created a File: Clinical Governance Registration Template which answers most of the common questions found in such documents.

Your local research and development team may wish to submit a separate registration via IRAS which can be linked to our project ID (338772) to automatically fill all of the relevant fields on the form. Our HRA approval letter and Organisation Information Document (OID), should they be requested, are available from the website (<https://www.uk-plan.net/REPACC>).

Early submission to your local research and development department is advised to prevent delay. Once approval is granted, please fill out the details of your sponsorship on the File: Organisational Survey CRF, send confirmation of your registration to [repacc.plan@gmail.com](mailto:repacc.plan@gmail.com) and proceed to step 1b.

**Step 1b**

## Organisational Survey

The organisational survey aims to capture the local setup of enhanced care at each site (File: Organisational Survey CRF). The questions require a reasonably in depth understanding of services offered and the structure of enhanced care locally. Information may be required from the medical and nursing leads for the following:

- Perioperative care.
- Enhanced recovery.
- Theatres.
- Post-anaesthetic care unit (PACU) / Overnight Intensive Recovery (OIR)
- Critical care.

The answer to each question should be entered into the right-hand column adjacent to the question stem. Certain question stems indicate the required format of the response, for example, (Yes/No) or (answer 1 to 4). If no guidance is given in the question stem the implied format is free text.

The process of answering this questionnaire will provide insight into the structure of local services and may help to identify sources of information for the following steps. Completion of this questionnaire may begin before research and development approval has been granted.

**Step 2a**

## Time-series of referrals to enhanced care

The first step in identifying your cohort, patients referred to enhanced care services pre-operatively between 01/09/23 and 30/11/23, is collating the list of referrals to each level of enhanced care during this period. This data may be stored in a multitude of different formats including secure email inboxes, physical referral diaries, locally designed databases, and electronic patient record (EPR) data. We will attempt to capture verbal and undocumented referrals in steps 3a and 3b.

As specified in the protocol, the referrals list should comprise adults (>18yrs) undergoing elective or expedited (NCEPOD 3 and 4) surgery – **excluding obstetric, cardiothoracic, and neurosurgical** procedures. This dataset should also exclude patients who have opted out of the use of their data for research purposes. It is unlikely that NCEPOD classification will be readily available for each patient, however, this is easy to work out. NCEPOD 4 represents planned elective procedures and will comprise the majority of the cohort. NCEPOD 3 represents expedited cases, these represent stable inpatients who require procedures within days of diagnosis, but the condition is not immediately life, limb, or organ threatening. Further information about this classification with examples can be found at <https://www.ncepod.org.uk/classification.html>.

In the case that patients had an enhanced care or critical care bed booked but their procedure was **not scheduled** to happen on that day please **exclude** these cases from the referrals column. Only if the procedure was scheduled to happen on the day of their planned admission, and this remained the case until the day of surgery, should they be included. For example, if somebody has proactively booked a level 2 bed for a patient undergoing a high risk vascular procedure, but 2 days before the date of surgery the procedure was rescheduled for another date exclude this case. Alternatively, if the same occurred on the day of surgery, please include this case as this represents a cancellation.

The hospital numbers of relevant referrals to each level of enhanced care should be entered into the File: Organisation Time-Series CRF. These should be entered into the three columns entitled “Hospital numbers of referrals to level X pre-operatively”. Each cell in these columns should contain a list of hospital numbers, **separated by a comma and a single space**, corresponding to the referrals to each level of enhanced care each day, **or left blank** if there were no referrals. The resultant series will resemble the following table:

| Date | Hospital numbers of referrals to level X pre-operatively |
| --- | --- |
| 01/09/23 | 123456, 234567, 345678 |
| 02/09/23 | 456789 |
| 03/09/23 |  |
| 04/09/23 | 567890, 678901 |
| 05/09/23 |  |
| 06/09/23 | 789012, 890123, 901234 |

[IMPORTANT] It is essential that hospital numbers are separated by a comma and a **single** space, and that days with no referrals are left blank, to permit automated counting of daily totals.

[IMPORTANT] If hospital numbers at your site contain a space in the middle, please omit this. For example, XY 1234 would become XY1234.

[TIMESCALE] Our pilot study found that it was possible to collate this data from a physical diary, with 0-8 referrals per day, within 1 afternoon. This may be longer for sites with busier units, however, it would be reasonable to schedule less than 1 week to complete this task.

**Step 2b**

## Time-series of operations, cancellations, and admissions

These data will be coded within surgical scheduling and bed management software at each site. To access this data, you will need to approach the clinical informatics or clinical coding team with formal governance approvals in place. This data will complete the remainder of the File: Organisation Time-Series CRF and represents the bulk of the data collection process. An example dataset that may be sent to your clinical informatics team for reference is provided in File: Time-Series Example Data. A request should be made for the following data.

#### Operations

These data should be collected as the sum-total of adult (>18yrs) procedures – excluding obstetric, cardiothoracic and neurosurgery – on each day of each category:

- Total number of operations performed between 01/09/23 and 30/11/23.
- Total number of **day case** procedures performed between 01/09/23 and 30/11/23.
- Total number of **non-emergency** (NCEPOD 3 and 4) procedures performed between 01/09/23 and 30/11/23.
- Total number of **emergency** procedures (NCEPOD 1 and 2) performed between 01/09/23 and 30/11/23.

#### Cancellations

- Hospital numbers of patients cancelled on-the-day for each day between 01/09/23 and 30/11/23.
- If a patient was rescheduled on the day of surgery then this also counts as a cancellation. (**On-the-day reschedule** = **Cancellation**)

Alongside hospital numbers, the reasons for cancellation may be valuable to request as these will be reliably linked to each event. These data are notoriously inaccurate but may serve as a prompt for the next steps where we identify those cancelled due to a lack of bed. On-the-day reschedules will likely represent the majority, with a minority being cancelled and not rescheduled.

Once collected, these should be recorded in the column “Hospital numbers of on-the-day cancellations” in File: Organisation Time-Series CRF. This should follow the same format as for referrals; **separated by a comma and a single space**.

#### Admissions to Level 1-3

Admissions data for each area should be collected in a way that captures the total resource use. Please apply the following criteria to each of the parameters in this section:

- Include all surgical admissions with no restrictions on type or urgency of surgery – the aim is to quantify total resource use.
- If several areas of the same level are present that admit surgical patients, please aggregate these data so that all admissions to each level on each day are present in a single cell.
- Admissions may include patients who were already admitted to this area that had further procedures and were re-admitted post-operatively.

#### Level 1

It will be necessary to identify which physical bedspaces or nursing unit represents your level 1 facility or facilities. It may be the case that certain bedspaces are only occasionally used for level 1 patients, perhaps as escalation. Please include these bedspaces as in the next steps it will be possible to eliminate those that were admitted to these beds but were considered level 0, or ward level, care. The following data should be requested:

- Hospital numbers of admissions to level 1 care area(s) **immediately** post-operatively (same day) between 01/09/23 and 30/11/23.

Once collected, these should be recorded in the column “Hospital numbers of admissions to level 1 immediately post-op” in File: Organisation Time-Series CRF. This should follow the same format as for referrals; **separated by a comma and a single space**.

#### Level 2-3

It will be necessary to identify which physical bedspaces or nursing unit represents your level 2-3 facilities. It may be the case that your level 2 and level 3 facilities share the same physical space, preventing reliable separation of level 2 and level 3 patients based on location alone. In this case, please collect aggregated admissions data for both areas in the level 3 columns and separate these out (if possible) in the next stages when looking at patient notes. The following data should be requested:

- Hospital numbers of admissions to level 2 care area(s) **immediately** post-operatively (same day) between 01/09/23 and 30/11/23.
- Hospital numbers of admissions to level 3 care area(s) **immediately** post-operatively (same day) between 01/09/23 and 30/11/23.
- Hospital numbers of admissions to level 2 care area(s) **within 7 days** post-operatively between 01/09/23 and 30/11/23.
- Hospital numbers of admissions to level 3 care area(s) **within 7 days** post-operatively between 01/09/23 and 30/11/23.

Once collected, these should be recorded in the columns in File: Organisation Time-Series CRF:

- Hospital numbers of admissions to level 2 immediately post-op
- Hospital numbers of admissions to level 2 of patients <7 days post-op
- Hospital numbers of admissions to level 3 immediately post-op
- Hospital numbers of admissions to level 3 of patients <7 days post-op

This should follow the same format as for referrals; **separated by a comma and a single space**. If any clarifications are required for any part of this dataset, please email [repacc.plan@gmail.com](mailto:repacc.plan@gmail.com) **excluding any patient identifiable information**.

[TIMESCALE] This process should take roughly 1-2 weeks. The clinical informatics and coding teams are often busy, so early contact once sponsorship is obtained is advisable.

**Step 3a**

## Cancellations due to a lack of enhanced care bed

The first method we will use to capture verbal and undocumented referrals is by looking at the list of cancellations from step 2b. In File: Organisation Time-Series CRF the column entitled “Hospital numbers of on-the-day cancellations” will include a list of hospital numbers of those cancelled or rescheduled on each day of the study period.

Open or request the notes for each of the patients in this list and appraise the reason for cancellation in each case. The coded reason for cancellation may have been collected in 2b and may be used as a guide, but this should not be relied on alone as these data are often inaccurate.

Hospital numbers of those that were cancelled due to a lack of enhanced care bed should be added to the column “Hospital numbers of on-the-day cancellations due to a lack of enhanced care bed”. Most of these hospital numbers should also appear in the column “Hospital numbers of referrals to level X pre-operatively”. If this is not the case, **these numbers should be added** **to the** “Hospital numbers of referrals to level X pre-operatively” **column**, as being cancelled for this reason implies that they were referred pre-operatively but that this was not recorded elsewhere.

**Step 3b**

## Admissions to enhanced care

The second method we will use to capture verbal and undocumented referrals is by looking at the list of admissions to each level of enhanced care from step 2b. In File: Organisation Time-Series CRF the following columns will include lists of hospital numbers of those admitted to enhanced care on each day of the study period:

- Hospital numbers of admissions to level 1 immediately post-op
- Hospital numbers of admissions to level 2 immediately post-op
- Hospital numbers of admissions to level 3 immediately post-op

Open or request the notes for each of the patients in these lists and identify elective admissions to each area that were not recorded in referral documentation. Elective admissions identified in this way should be added to the corresponding “Hospital numbers of referrals to level X pre-operatively” column.

________________________________________________________________________________

[IMPORTANT] If you have included beds that are occasionally used to host enhanced care patients in your sample it will be necessary to remove the hospital numbers of those admitted for level 0 care only from the admissions list for each area.

[IMPORTANT] If it was only possible to collect aggregate admissions data in step 2b here it will be necessary to identify which level of care each patient was admitted for from their notes or using SitRep/ICNARC data.

[IMPORTANT] **At sites with paper notes only**, steps 3a and 3b will require requesting the notes of each patient that was cancelled or admitted to enhanced care during the study period. In this situation, the data collection process is likely to require more team members and more time to complete the task. Patients that are identified as having been referred to enhanced care pre-operatively through these methods, alongside those from referral documentation, will comprise your study cohort. Therefore, it would be sensible to retain the notes for the per-patient data collection steps that will follow, or indeed perform step 5 in tandem with steps 3a and 3b.

**Step 4**

## Consolidation of final cohort

The following columns in File: Organisation Time-Series CRF will include patients that were referred to enhanced care pre-operatively:

- Hospital numbers of referrals to level 1 pre-operatively
- Hospital numbers of referrals to level 2 pre-operatively
- Hospital numbers of referrals to level 3 pre-operatively

These should be consolidated into a final cohort in File: Patient CRF. Each patient should represent a single row. The Excel tab entitled EhC Referrals will contain an aggregated list of referrals to all levels of enhanced care with linked study IDs (SIDs). This list can be copied directly into File: Patient CRF if you have not constructed this list manually and can be used for cross referencing.

[TIMESCALE] Steps 3-4 represent the bulk of the data collection. Depending on the number of cancellations and admissions, and whether your organisation uses paper notes, it would be sensible to schedule 2-3 weeks to complete these steps.

**Step 5a**

## Patient level coding data

Return the list of hospital numbers on File: Patient CRF to your clinical informatics or clinical coding team and request the following data for each patient. Many of the fields require a particular format, please check this format before collecting and perform the necessary transformations.

Data type is enclosed in square brackets.

- [bool] = boolean/True:False, denoted as 1 for True and 0 for False
- [datetime] = date & time
- [int] = integer, number
- [str] = string/text

#### Demographics

- Age [int]
- Gender
  - Gender male [bool]
  - Gender female [bool]
  - Gender non-binary [bool]
- Ethnicity.
  - Asian or Asian British [bool]
  - Black, Black British, Caribbean or African [bool]
  - Mixed or multiple ethnic groups [bool]
  - White [bool]
  - Other ethnic group [bool]

#### Comorbidities

- Coded comorbidities.
  - Chronic cardiac disease [bool]
  - Chronic kidney disease [bool]
  - Chronic liver disease [bool]
  - Chronic neurological disease [bool]
  - Chronic pulmonary disease [bool]
  - Dementia [bool]
  - Diabetes [bool]
  - Diabetes with chronic complication [bool]
  - Hypertension [bool]
  - Malignancy [bool]
  - Metastatic malignancy [bool]
  - Peripheral vascular disease [bool]
  - Rheumatic disease [bool]

#### Planned procedure details

- Primary surgical speciality [str]
- Proposed surgical procedure [str]
- Cancer surgery flag [bool]
- NCEPOD classification [int]
- Operative severity (Minor-Complex) [str] – this is derived from AXA procedure codes which are used for costing in most Trusts, a full list of linked procedure codes is available from the AXA website (<https://specialistforms.onlineapps.axahealth.co.uk/SpecialistCode.mvc/Print?source=contracted>) and in .csv format from the SNAP-2 study documentation (<https://www.rcoa.ac.uk/sites/default/files/documents/2022-10/SNAP-2%20Procedure%20List.xlsx>)
- Scheduled date and time of procedure [datetime]
- Previous cancellation flag [bool]

#### Procedure outcomes

- Knife to skin time of procedure [datetime]
- Procedure cancelled flag [bool]
- Post-op critical care within 7 days flag [bool]
- Encounter start date and time [datetime]
- Encounter end date and time [datetime]
- Length of stay encounter [int] – this is calculated by the CRF
- Length of stay level 0 [int]
  - Total days in this nursing unit during this admission
- Length of stay level 1 [int]
  - Total days in this nursing unit during this admission
- Length of stay level 2 [int]
  - Total days in this nursing unit during this admission
- Length of stay level 3 [int]
  - Total days in this nursing unit during this admission
- Mortality flag [bool]
- Mortality date and time [datetime]

**Step 5b**

## Patient level collected data

This final stage of data collection may be performed in tandem with steps 3a and 3b, especially in centres with paper-based notes. There is minimal data to collect for each patient and therefore the rate limiting step will be accessing their notes. The following should be collected for each patient in your cohort and input into File: Patient CRF. Boolean [bool] values should be filled with a “1” to indicate “True” and a “0” to indicate “False”. The file will not accept any values that are not 0 or 1 for Boolean fields.

#### Cancellation

Only complete this section if the patient had their operation cancelled. Otherwise, leave these cells blank.

- Cancellation due to a lack of enhanced care bed flag [bool]
- Cancelled due to other reason flag [bool]
- Other reason for cancellation [str]
  - Free text reason for cancellation.

#### Referral

This section aims to capture the process of referral to enhanced care for each patient in terms of timing and rationale.

Timing:

- Referral 8 days or more before flag [bool]
  - e.g. Referred on Tuesday 03/11/23, surgery scheduled to be performed on Wednesday 11/11/23 (=8 days).
- Referral 1-7 days before flag [bool]
  - e.g. Referred on Wednesday 04/11/23, surgery scheduled to be performed on Wednesday 11/11/23 (=7 days).
  - e.g. Referred on Monday 09/11/23, surgery scheduled to be performed on Wednesday 11/11/23 (=2 days).
- Referral on the day flag [bool]

Rationale:

- Part of SOP for surgery flag	[bool]
  - For example, this should include all laparotomies. It will be necessary to obtain your local protocol, pathways document or policy that specifies which operations incur an admission to enhanced care as standard.
- CPET result flag [bool]
- Clinical judgement flag [bool]
- Risk score flag [bool]
- Risk score name [str]
  - Free text name of risk score used.
- Predicted mortality [int]
  - Numerical value (**15% => 0.15**) of the percentage predicted mortality, according to the score used, if available.
- Predicted morbidity [int]
  - Numerical value (**15% => 0.15**) of the percentage predicted morbidity, according to the score used, if available.
- Referred for other reason flag [bool]
- Other reason for referral [str]
  - Free text rationale for referral.

#### Escalation or de-escalation of care

This section aims to capture the journey of each patient in the immediate post-operative period. Much of this data will already be available in File: Organisation Time-Series CRF.

- Proposed post-operative critical care level (1-3) [int]
- Actual post-operative critical care level (0-3) [int]
- Difference due to lack of enhanced care bed flag [bool]
  - If there was an equal number of admissions to the proposed area as there are ring-fenced beds on the day a patient was “de-escalated” then it would be appropriate to identify this as the reason.
- Difference for other reason flag [bool]
- Other reason for difference [str]
  - Free text reason for difference between proposed and actual post-operative destination if available.
- Reason for difference unknown flag [bool]

[TIMESCALE] This final stage of data collection should take roughly 1 week to complete. This may be performed in tandem with steps 3a and 3b.

# Submission

To limit any potential harm caused by sharing of information outside of the immediate care team study IDs are automatically generated for each patient. When sharing data with regional research teams or the study management group (SMG) please do **not** include any hospital numbers or directly identifiable patient information, such as name or date of birth.

Advice concerning submission of each individual CRF is as follows:

- File: Organisational Survey CRF:
  - Submit this as a file entitled Organisational Survey CRF followed by your Site Code.
- File: Organisation Time-Series CRF:
  - Firstly, please ensure that all of the green boxes in Tab: SID Linked and Tab: Anonymised Data have not turned red, these are quality assurance markers which indicate if there are typos or inconsistencies in the dataset. If there are errors, please return to the relevant section of Tab: Input Data and amend the error before submission (this will most likely be an extra space or comma placed somewhere).
  - Once this is completed, copy the entirety of Tab: Anonymised Data and paste it into a new Excel workbook entitled Organisation Time-Series CRF followed by your Site Code.
  - Perform a final check to ensure that no hospital numbers have been included in this file and submit.
- File: Patient CRF:
  - Create a copy of this file entitled Patient CRF followed by your Site Code.
  - In this version, delete the first column containing hospital numbers. Please ensure that study IDs (SIDs) are retained.
  - Perform a final check to ensure that no hospital numbers or personally identifiable information have been included in this file and submit.

Please submit the completed CRFs to: [chris.oddy@nhs.net](mailto:chris.oddy@nhs.net)

**Please do not submit any patient information to the REPACC Gmail address as this is not a secure mailbox.** Local leads are responsible for the appropriate stewardship of sensitive information prior to submission to the SMG.
