# Supplementary material for: Surgical cancellation and changes in postoperative level of care among high and moderate risk surgical patients in the UK
Source: J Intensive Care Soc. 2026 Oct 12:17511437261495043. Online ahead of print. doi: 10.1177/17511437261495043 (PMC13658631; doi:10.1177/17511437261495043)
Supplement: Supplementary file 5 [file sj-docx-6-inc-10.1177_17511437261495043.docx]

**Appendix E**	PLAN committee members involved in delivery of the REPACC study.

- Dr Matthew Sinnott
- Dr Mayur Murali
- Dr Shriya Kumar
- Dr Farzana Husain
- Dr Muska Miller
- Dr Elizabeth Brown
- Dr Jan Man Wong
- Dr Kim Rhodes
- Dr Lucy Liu
- Dr Daveena Meeks
- Dr Ben Milne
- Dr Jenny Olsson
- Dr Mitul Patel
- Dr Shilen Shah
