# Supplementary material for: Surgical cancellation and changes in postoperative level of care among high and moderate risk surgical patients in the UK
Source: J Intensive Care Soc. 2026 Oct 12:17511437261495043. Online ahead of print. doi: 10.1177/17511437261495043 (PMC13658631; doi:10.1177/17511437261495043)
Supplement: Supplementary file 6 [file sj-docx-7-inc-10.1177_17511437261495043.docx]

**Table A1**	A comparison of the demographics, comorbidities, and primary surgical specialities of patients whose surgery was cancelled for different reasons. Values are median (IQR) for age and otherwise counts and percentages.

|  | **All cancellations**  **n = 539** | **Lack of resources**  **n = 275** | **Clinical reasons**  **n = 153** | **Personal reasons**  **n = 26** | **Administrative**  **n = 7** | **Unknown reason**  **n = 78** |
| --- | --- | --- | --- | --- | --- | --- |
|  |  |  |  |  |  |  |
| Median age | 66 (55-76) | 65 (51-75) | 68 (58-78) | 70.5 (63-78) | 63 (40-70) | 65 (55-74) |
| Male | 307 (57.0%) | 157 (57.1%) | 81 (52.9%) | 18 (69.2%) | 6 (85.7%) | 45 (57.7%) |
| White ethnicity | 412 (76.4%) | 215 (78.2%) | 123 (80.4%) | 16 (61.5%) | 5 (71.4%) | 53 (67.9%) |
|  |  |  |  |  |  |  |
| **Comorbidities** |  |  |  |  |  |  |
| Chronic cardiac disease | 212 (39.3%) | 107 (38.9%) | 69 (45.1%) | 9 (34.6%) | 4 (57.1%) | 23 (29.5%) |
| Chronic kidney disease | 89 (16.5%) | 43 (15.6%) | 35 (22.9%) | 3 (11.5%) | 1 (14.3%) | 7 (9.0%) |
| Chronic liver disease | 42 (7.8%) | 15 (5.5%) | 12 (7.8%) | 3 (11.5%) | 1 (14.3%) | 11 (14.1%) |
| Chronic neurological disease | 82 (15.2%) | 46 (16.7%) | 23 (15.0%) | 5 (19.2%) | 0 (0.0%) | 8 (10.3%) |
| Chronic pulmonary disease | 163 (30.2%) | 88 (32.0%) | 43 (28.1%) | 7 (26.9%) | 1 (14.3%) | 24 (30.8%) |
| Dementia | 10 (1.9%) | 7 (2.5%) | 2 (1.3%) | 1 (3.8%) | 0 (0.0%) | 0 (0.0%) |
| Diabetes | 136 (25.2%) | 71 (25.8%) | 34 (22.2%) | 7 (26.9%) | 2 (28.6%) | 22 (28.2%) |
| Hypertension | 267 (49.5%) | 134 (48.7%) | 78 (51.0%) | 17 (65.4%) | 2 (28.6%) | 36 (46.2%) |
| Malignancy | 196 (36.4%) | 91 (33.1%) | 71 (46.4%) | 8 (30.8%) | 3 (42.9%) | 23 (29.5%) |
| Metastasis | 44 (8.2%) | 19 (6.9%) | 18 (11.8%) | 2 (7.7%) | 0 (0.0%) | 5 (6.4%) |
| Peripheral vascular disease | 60 (11.1%) | 26 (9.5%) | 20 (13.1%) | 0 (0.0%) | 2 (28.6%) | 12 (15.4%) |
| Rheumatological disorders | 34 (6.3%) | 19 (6.9%) | 6 (3.9%) | 1 (3.8%) | 0 (0.0%) | 8 (10.3%) |
|  |  |  |  |  |  |  |
| **Surgical specialty performing procedure** |  |  |  |  |  |  |
| General surgery | 163 (30.2%) | 91 (33.1%) | 50 (32.7%) | 9 (34.6%) | 0 (0.0%) | 13 (16.7%) |
| Gynaecological surgery | 23 (4.3%) | 12 (4.4%) | 7 (4.6%) | 1 (3.8%) | 0 (0.0%) | 3 (3.8%) |
| Hepatobiliary surgery | 60 (11.1%) | 33 (12.0%) | 14 (9.2%) | 3 (11.5%) | 2 (28.6%) | 8 (10.3%) |
| Orthopaedic surgery | 91 (16.9%) | 48 (17.5%) | 23 (15.0%) | 4 (15.4%) | 0 (0.0%) | 16 (20.5%) |
| Otorhinolaryngology, maxillofacial and head & neck surgery | 38 (7.1%) | 20 (7.3%) | 8 (5.2%) | 2 (7.7%) | 2 (28.6%) | 6 (7.7%) |
| Plastic surgery | 2 (0.4%) | 1 (0.4%) | 1 (0.7%) | 0 (0.0%) | 0 (0.0%) | 0 (0.0%) |
| Renal & urological surgery | 68 (12.6%) | 30 (10.9%) | 28 (18.3%) | 2 (7.7%) | 1 (14.3%) | 7 (9.0%) |
| Vascular surgery | 56 (10.4%) | 27 (9.8%) | 15 (9.8%) | 4 (15.4%) | 1 (14.3%) | 9 (11.5%) |
| Unspecified | 38 (7.1%) | 13 (4.7%) | 7 (4.6%) | 1 (3.8%) | 1 (14.3%) | 16 (20.5%) |
|  |  |  |  |  |  |  |
