# Supplementary material for: Surgical cancellation and changes in postoperative level of care among high and moderate risk surgical patients in the UK
Source: J Intensive Care Soc. 2026 Oct 12:17511437261495043. Online ahead of print. doi: 10.1177/17511437261495043 (PMC13658631; doi:10.1177/17511437261495043)
Supplement: Supplementary file 7 [file sj-docx-8-inc-10.1177_17511437261495043.docx]

**Table B1**	A comparison of the demographics, comorbidities, and primary surgical specialities of patients were escalated and de-escalated to different levels of care postoperatively. Values are median (IQR) for age and otherwise counts and percentages.

|  | **Overall**  **n = 1019** | **Escalations**  **n = 210** | **De-escalations**  **n = 809** |
| --- | --- | --- | --- |
|  |  |  |  |
| Median age | 67 (56.0-75.0) | 69 (56.0-75.0) | 66 (56.0-75.0) |
| Male | 545 (53.5%) | 128 (61.0%) | 417 (51.5%) |
| White ethnicity | 792 (77.7%) | 163 (77.6%) | 629 (77.8%) |
|  |  |  |  |
| **Comorbidities** |  |  |  |
| Chronic cardiac disease | 286 (28.1%) | 70 (33.3%) | 216 (26.7%) |
| Chronic kidney disease | 153 (15.0%) | 36 (17.1%) | 117 (14.5%) |
| Chronic liver disease | 64 (6.3%) | 14 (6.7%) | 50 (6.2%) |
| Chronic neurological disease | 93 (9.1%) | 19 (9.0%) | 74 (9.1%) |
| Chronic pulmonary disease | 277 (27.2%) | 53 (25.2%) | 224 (27.7%) |
| Dementia | 13 (1.3%) | 2 (1.0%) | 11 (1.4%) |
| Diabetes | 205 (20.1%) | 48 (22.9%) | 157 (19.4%) |
| Hypertension | 437 (42.9%) | 98 (46.7%) | 339 (41.9%) |
| Malignancy | 478 (46.9%) | 108 (51.4%) | 370 (45.7%) |
| Metastasis | 98 (9.6%) | 23 (11.0%) | 75 (9.3%) |
| Peripheral vascular disease | 88 (8.6%) | 22 (10.5%) | 66 (8.2%) |
| Rheumatological disorders | 104 (10.2%) | 20 (9.5%) | 84 (10.4%) |
|  |  |  |  |
| **Surgical specialty performing procedure** |  |  |  |
| General surgery | 426 (41.8%) | 77 (36.7%) | 349 (43.1%) |
| Gynaecological surgery | 76 (7.5%) | 13 (6.2%) | 63 (7.8%) |
| Hepatobiliary surgery | 90 (8.8%) | 26 (12.4%) | 64 (7.9%) |
| Orthopaedic surgery | 112 (11.0%) | 32 (15.2%) | 80 (9.9%) |
| Otorhinolaryngology, maxillofacial and head & neck surgery | 49 (4.8%) | 12 (5.7%) | 37 (4.6%) |
| Plastic surgery | 9 (0.9%) | 2 (1.0%) | 7 (0.9%) |
| Renal & urological surgery | 112 (11.0%) | 12 (5.7%) | 100 (12.4%) |
| Vascular surgery | 91 (8.9%) | 33 (15.7%) | 58 (7.2%) |
| Unspecified | 54 (5.3%) | 3 (1.4%) | 51 (6.3%) |
|  |  |  |  |
